# Can the co-cultivation of rice and fish help sustain rice production?

**DOI:** 10.1038/srep28728

**Published:** 2016-06-28

**Authors:** Liangliang Hu, Jian Zhang, Weizheng Ren, Liang Guo, Yongxu Cheng, Jiayao Li, Kexin Li, Zewen Zhu, Jiaen Zhang, Shiming Luo, Lei Cheng, Jianjun Tang, Xin Chen

**Affiliations:** 1 College of Life Sciences, Zhejiang University, Hangzhou 310058, China; 2 College of Aquaculture and Life Sciences, Shanghai Ocean University, Shanghai 201306, China; 3 National Aquaculture Technical Extension Station, Ministry of Agriculture of People’s Republic of China, Beijing 100125, China; 4 Department of Ecology, South China Agricultural University, Guangzhou 510642, China

## Abstract

Because rice feeds half of the world’s population, a secure global food supply depends on sustainable rice production. Here we test whether the co-cultivation of rice and fish into one “rice-fish system” (RFS; fish refers to aquatic animals in this article) could help sustain rice production. We examined intensive and traditional RFSs that have been widely practiced in China. We found that rice yields did not decrease when fish yield was below a threshold value in each intensive RFS. Below the thresholds, moreover, fish yields in intensive RFSs can be substantially higher than those in traditional RFS without reducing rice yield. Relative to rice monoculture, the use of fertilizer-nitrogen and pesticides decreased, and the farmers’ net income increased in RFSs. The results suggest that RFSs can help sustain rice production, and suggest that development of co-culture technologies (i.e. proper field configuration for fish and rice) is necessary to achieve the sustainability.

Rice is the main component in the daily diets of about 3 billion people^1^, many of whom live in developing areas and cannot afford high prices for rice^2^. Future global food security and the precarious livelihoods of the world’s poor depend on a reliable supply of rice. How to meet the increasing demand for rice without increasing environmental cost is a great challenge for the world rice farming^2^.

Rice fields can provide habitat for “fish” (the term ‘fish’ in this article refers to a wide range of aquatic animals including carp, crab, crayfish, soft-shelled turtle, and others)^3^,^4^. The coupling of rice culture with fish production (also referred to as a rice–fish system or an RFS) has long been practiced in many rice-growing areas because it provides both rice grain and an aquatic protein source^3^,^4^. Because raising fish in rice fields can often reduce the use of pesticides for rice by reducing the incidence of diseases, insect pests, and weeds, and can also reduce the use of chemical fertilizer-nitrogen (N) through the complementary use of feed-N between rice and fish, RFSs have been considered an important way to help sustain local rice farming^5^,^6^,^7^,^8^.

The Food and Agriculture Organization of the United Nation and other international organizations have been attempting to develop RFSs in the rice-growing areas of the world in order to help secure food supplies and improve rural economies^5^,^6^,^9^. Researchers recently proposed that RFSs could be useful for expanding freshwater aquaculture and increasing water productivity in some Asian countries (e.g., Bangladesh and Indonesia)^10^,^11^. In China, the development of RFSs has also been listed as a national strategy to simultaneously produce rice and freshwater fish^12^,^13^. The Ministry of Agriculture of the People’s Republic of China has been trying to help farmers improve traditional RFSs and develop intensive RFSs with high fish yields^12^,^13^ in order to increase rice-field productivity and farmer income. Since 2010, China has had the largest RFS area in the world^12^,^13^.

Traditional RFSs use only small amounts of fish feed and keep the fish in only a small area that is referred to as a “fish refuge”; as a consequence, fish yields in traditional RFSs are relatively low, and rice yield is not reduced^5^,^7^,^14^. Intensive RFSs, in contrast, are large-scale operations that use relatively high quantities of commercial “fish” feed and large fish refuges to achieve high fish yields and significant farmer profits^12^,^13^. This suggests two important and related questions regarding intensive RFSs. Are rice yields lower in intensive RFSs than in rice monoculture? Can the intensive RFSs help sustain rice production? From the perspective of national food security, it is essential to determine whether the development of intensive RFSs threatens rice production.

In this study, we undertake a survey to assess rice yields in various types of RFSs across five rice-growing areas with different climates in China. We discuss the implications of our results for the development and management of intensive RFSs with the goal of sustaining rice farming in China and other countries.

Because the raising of fish in rice fields could promote rice growth and benefit rice yield, and because the raising of fish requires that some percentage of each rice field be devoted to a fish refuge, we expected that rice yield could change in response to changes in fish yield and that rice yield might be reduced if fish yields exceeded some threshold. We therefore hypothesized that there is some maximum level of fish yield at which rice yield is not harmed. We tested this hypothesis with a farm and farmer survey that included one type of traditional RFS (rice-carp) and five types of intensive RFSs (rice-carp, rice-crab, rice-crayfish, rice-loach, and rice-turtle [soft-shelled turtle]). The survey covered 56 counties in 14 provinces or cities (Fig. 1a). A traditional RFS is defined here as a small-scale farming system without application of commercial fish feed, and an intensive RFS is defined as a relative large-scale farming system with application of relatively high quantities of commercial fish feed.

## Results

**Figure 1:**
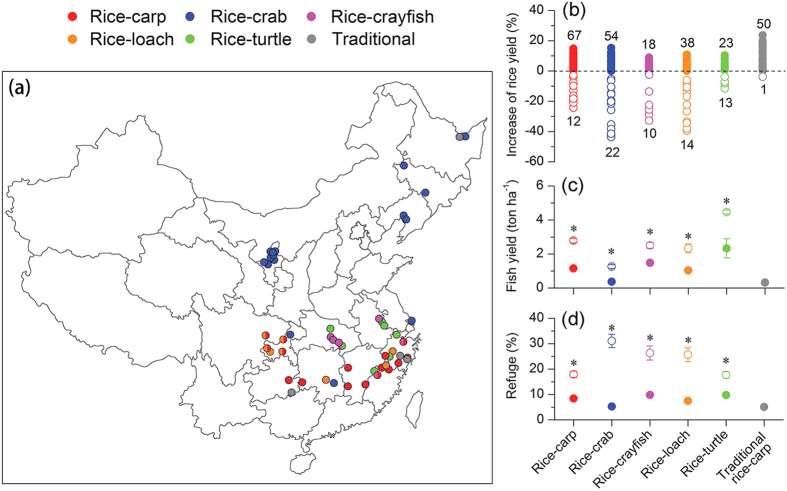
Performance of rice and fish yields in the sampled traditional and intensive RFSs. **(a)** Survey sites. The dots indicate survey sites at the county level, and dot colour indicates the kind of RFSs, as indicated by the legend. The map was modified from the map of People’s Republic of China that was provided at the free standard map service by the National Administration of Surveying, Mapping and Geoinformation of China (http://219.238.166.215/mcp/Default.html). **(b)** Changes in rice yield in RFSs relative to their corresponding RMs. Rice yield change (%) = ([yield in RFS-yield in RM]/yield in RM) × 100%. Numbers at the top (filled dots) and bottom (hollow dots) for each type of RFS indicate the number of RFS farms with not decreased yield or decreased yield, respectively, relative to the corresponding RM farm. **(c)** Mean fish yields in RFSs where rice yield decreased (hollow dots) or did not decrease (filled dots). **(d)** Refuge percentages in RFSs where rice yield decreased (hollow dots) or did not decrease (filled dots). In (**c**,**d**), *indicates a significant difference (P < 0.05).

Rice yield was lower in intensive RFSs than in the paired RMs for 15% of the rice-carp farms, 29% of the rice-crab farms, 36% of the rice-crayfish farms, 27% of the rice-loach farms, and 27% of the rice-turtle farms (Fig. 1b). For 98% of the traditional rice-carp farms, however, rice yield was not lower than in the corresponding RM farms (Fig. 1b). Further analysis revealed that fish yield and refuge percentage were significantly higher in RFS farms where rice yield had decreased relative to RM than in RFS farms where rice yields had not decreased relative to RM farms (P < 0.05, Fig. 1c,d).

**Figure 2:**
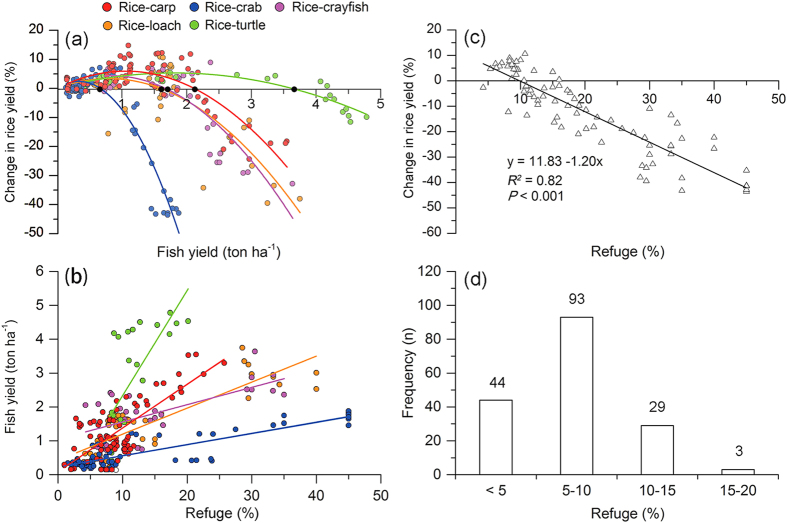
Relationship between change in rice yield (%) and fish yield, and between yields (rice, fish) and refuge percentage in the intensive RFSs. (**a**) The relationship between change in rice yield (%) and fish yield for each type of intensive RFS. The black dots along the X axis indicate the maximum fish yields (the thresholds) that did not result in decreases in rice yield. The threshold values were estimated by curve fitting data for change in rice yield (%) vs. fish yield (see Supplementary Table 1). (**b**) Fish yield as a function of the percentage of the field used as a refuge. (**c**) The relationship between change in rice yield (%) and refuge area (%) when fish yield exceeded the thresholds indicated in (**a**). (**d**) The sample size of each group of refuge area (%).

As expected, there was a threshold value for fish yield for each type of intensive RFS. Rice yields in intensive RFSs were less than those in paired RMs when fish yields exceeded the following threshold values (in ton ha^−1^): 2.106 (95% CI:2.101, 2.112) for rice-carp; 0.657 (95% CI: 0.653, 0.661) for rice-crab; 1.681 (95% CI: 1.671, 1.691) for rice-crayfish; 1.582 (95% CI:1.571, 1.593) for rice-loach; and 3.621 (95% CI:3.613, 3.628) for rice-turtle (Fig. 2a, Supplementary Table 1). Our results also showed that fish yield continuously increased as the percentage of the field used as a refuge (refuge percentage) increased for each type of intensive RFSs (Fig. 2b, Supplementary Table 2). The slopes of linear relationship between aquatic yield with refuge percentage were significantly different among the RFSs (F_3,167_ = 38.155, P < 0.001). The slope of linear relationship in rice-turtle was significantly higher than in other RFSs, while there was no significant difference of slope between rice-crab and rice crayfish, and between rice-carp and rice-loach (Supplementary Table 2). When fish yields exceeded the threshold values identified in Fig. 2(a), however, rice yield was negatively related to refuge percentage (Fig. 2c). The results also indicated that in the overwhelming majority of cases where fish yield was lower than the threshold for rice yield not decrease, the area of refuge was less than 15%, and in most cases less than 10% (Fig. 2d).

**Figure 3:**
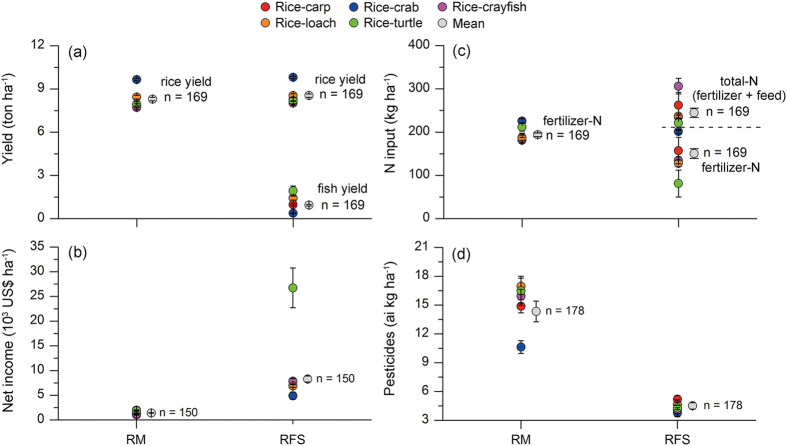
Output and input in the intensive RFS farms where rice yields were below the threshold (see Fig. 2a) and in their corresponding RM farms. (**a**) Yields of rice and fish. (**b**) Total net income. (**c**) Fertilizer-N. (**d**) Pesticides. Values are mean ± SE.

**Figure 4:**
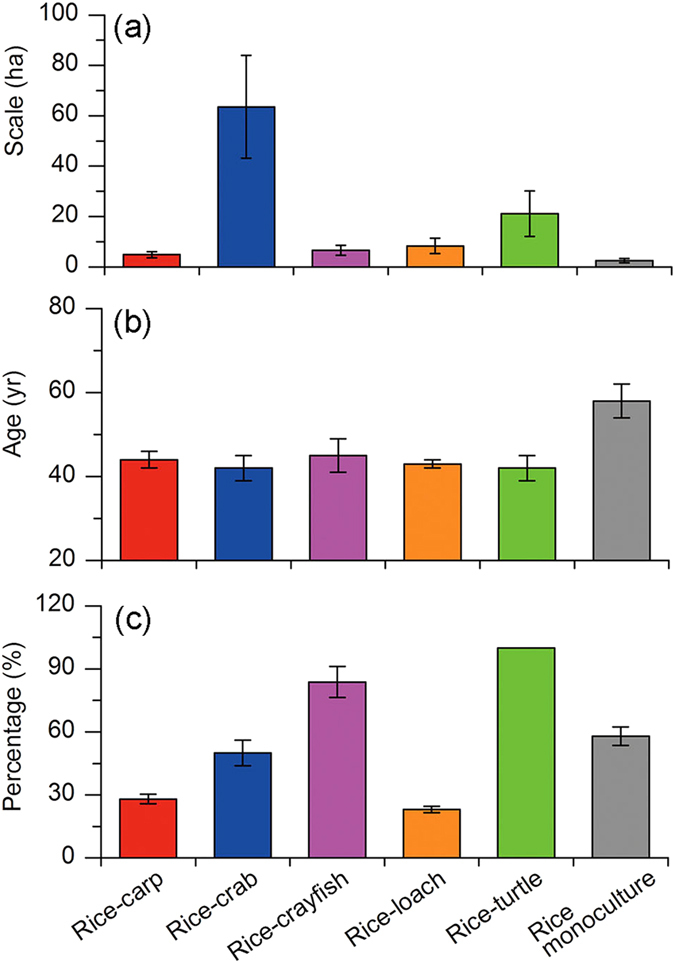
Some characteristics of intensive RFS farms and rice monoculture farms. (**a**) Farm size; (**b**) Farmer age; and (**c**) Percentage of farms managed by specialized farmer cooperatives.

For intensive RFS farms where fish yields were below the thresholds, rice yields were equivalent to those in corresponding RMs in the case of rice-turtle and rice-loach RFSs (P > 0.05) or were higher than those in corresponding RMs in the case of rice-carp, rice crab, and rice-crayfish RFSs (P < 0.05). Moreover, these intensive RFSs produced an average fish yield (in ton ha^−1^) of 0.97 for rice-carp, 0.387 for rice-crab, 1.437 for rice-crayfish, 1.097 for rice-loach, and 1.977 for rice-turtle (Fig. 3a, Supplementary Table 3). These RFSs received higher total N input (feed-N plus fertilizer-N) than RMs, but they used less fertilizer-N and pesticides than RMs (P < 0.05, Fig. 3c,d, Supplementary Table 3). The prices obtained for grain and fish from these RFSs were higher than from local non-RFS farms (Supplementary Table 4), and net income was higher for these RFS farmers than for RMs farmers (Fig. 3b, Supplementary Table 3). Relative to RM farms, intensive RFS farms were larger, were more likely to belong to specialized farmer cooperatives, and were managed by younger farmers (Fig. 4a,c).

## Discussion

Our results revealed that rice yields were severely decreased when the large field areas were used as refuges to increase fish yield in intensive RFSs. Our results also indicated that each type of intensive RFS had a threshold of fish yield below which rice yield was not reduced even though about 10% of the field area was used as a refuge; the refuge percentage in the traditional rice-carp RFS in this study was <5%. Below the threshold, rice yield in intensive RFSs is not suppressed because fish enhance the growth of the remaining rice plants^6^ and because the refuges (trenches and pits) create an “edge effect” for the growth of rice plants. As long as the threshold for fish yield is not exceeded and the refuge occupies <10% of the field, the combined effects of fish and refuge edges can compensate or even over-compensate for the loss of plants due to refuge building^5^,^15^. When farmers use a high percentage of the area for refuges (famers sometimes use up to 50% when the price of fish is substantially higher than that of rice), however, rice production will certainly be reduced (Fig. 2c). Our study also indicated that the refuge areas differed in effecting fish yield among RFSs. In rice-turtle system, for example, fish yield increased greatly as the increase of refuge percentage. In rice-crab or rice-crayfish, however, fish yield increased slowly with the increase of refuge percentage (Fig. 2b, Supplementary Table 2). These results suggest that refuge design is important for both rice and fish yield in the development of RFSs.

Our results suggest that the current intensive RFSs can help sustain rice farming when the trade-off between rice and fish yield is balanced. On the one hand, rice yield in RFSs did not decrease and in some cases even increased when fish yield was below the threshold. This threshold for fish yield for each intensive RFS was significantly higher than the fish yield in the traditional rice-carp RFS (0.33 ton ha^−1^), indicating that fish yield in intensive RFSs can be substantially higher than those in traditional RFSs without reducing rice yield. On the other hand, RFSs can stimulate farmer enthusiasm for rice farming. First, RFS farmers produce both rice grain and fish, and the latter usually has a high market price resulting in increased income. Second, fertilizer-N (Fig. 3c) and pesticide (Fig. 3d) are significantly lower in RFSs than in RM because fish can help remove rice pests and because rice can use the unconsumed feed-N^7^,^16^. The reduction of fertilizer-N and pesticide reduces the cost of rice farming and also leads to the marketing of identifiable brands of high quality rice or fish products. Both rice and fish products with identifiable brands were accepted by consumers and obtained higher prices than standard products (Supplementary Table 3). Thus, intensive RFSs can greatly increase farmer income (Fig. 3b), and this increases the interest of young farmers in RFSs. According to our farmer survey, 57% of RFSs are managed by specialized large-scale farmer cooperatives, which are led by young adults (Fig. 4).

From the point of economic rationalist, however, one may worry whether the high income from fish in RFSs would lead farmers to overemphasize fish yield and its income and ignore rice that will threaten rice production. In our study, we did find some cases in each type of RFSs that farmers increase the production of fish by sacrifice rice yields (Fig. 2a). In most cases, however, the economic rationalist approach is not necessarily the optimal pathway in China. First, rice is not a purely economic crop because rice is one of the most important crops needed for national food security. Rice production is partially regulated by government policy. For example, a guideline for securing rice yield in RFSs has been established by the National Aquaculture Technical Extension Station of the Ministry of Agriculture of China^12^ for helping farmers to develop RFSs avoiding the decrease of rice yield in RFSs. Second, higher fish yields from RFSs require large refuge areas (Fig. 2b), resulting in the decrease of rice plant density. As rice plants can use the nutrients (i.e. N and phosphorus) releasing from the unconsumed- feed or fish excrement^7^,^13^, the decrease of rice plant density would result in low nutrient uptake by rice plants and the accumulation of nutrients in the rice field. Moreover, higher fish yields require higher feed input, from which only 20–30% of N was assimilated by fish^7^,^17^. Thus, there are increased environmental risks (e.g. nutrient discharge) in RFSs when fish yields exceed a particular level for a long time.

Rice farming in China and in other countries is facing the challenge of sustaining rice yield without increasing the use of fertilizer-N, pesticides, and other costly inputs and the challenge of promoting farmer enthusiasm for rice farming^2^,^18^,^19^. Our results demonstrated that intensive RFSs can increase aquatic food supply and farmer income and can help sustain rice production. These results also provide referential experiences for several other countries (e.g. Egypt, India, Indonesia, Thailand, Vietnam, the Philippines, Bangladesh, Myanmar, and Malaysia), which are practicing RFSs^8^,^10^. As rice and aquatic food are essential components of local diets in many of these countries, given the millions of hectares of rice field with suitable conditions, increase in RFS area would help sustain the world food supplies.

Achieving acceptable and sustainable levels of rice and fish yields in these intensive RFSs, however, requires a co-culture technology package that is much more sophisticated than that required for either rice or fish monoculture. This package includes proper field configuration for rice and fish, varieties of rice and fish adapted to rice and fish co-culture, suitable machinery, and information-based integrated daily field management^13^,^14^. The establishment and implementation of this package also requires policy support and coordination across governmental departments^12^,^13^,^20^.

## Methods

### Types of RFSs considered in this study

The one traditional rice-carp and five intensive RFSs (rice-carp, rice-crab, rice-crayfish, rice-loach, and rice-turtle, Supplementary Figure, Supplementary Panel 1) considered in this study are the most important types of RFSs in China, where they account for about 80% of the total area of RFSs (2.32 million ha annually)^21^. The five intensive RFSs were developed and adapted to rice production throughout the five rice-planting areas of China and have helped supply fish protein to local people^13^. Compared to the traditional rice-carp RFS, these five intensive RFSs are managed to produce higher fish yields as described in Supplementary Panel 1. All the farm surveys involving “fish” were carried out in accordance with the approved guidelines of Zhejiang University Experimental Animal Management Committee.

### Farm survey

A total of 309 paired farms, i.e., RFS vs. rice monoculture (RM), were surveyed in 2014 in the main rice-growing areas of China. The paired farms included 51 traditional rice-carp RFS and the following numbers of intensive RFSs: 79 with rice-carp, 76 with rice-crab, 28 with rice-crayfish, and 52 with rice-loach, 23 with rice-turtle. The survey included 56 counties in 14 provinces or cities (Fig. 1a). Each pair of RM and RFS farms was located in the same village and had a similar climate and soil condition. The farm pairs were owned by one farmer or by two neighbouring farmers. Rice and fish yields for each farm were determined by using the data collected by the farmer at harvest. Rice yield was measured as air-dried weight, and fish yield was measured as fresh weight. Rice and fish yields are expressed as ton ha^−1^. The survey was also used to determine the percentage of each field used as a fish refuge (refuge percentage).

We also recorded the rice varieties and irrigation used in each field. All applications of nitrogen (N) fertilizer, pesticide, and fish feed on each farm were recorded during the rice- and fish-growing season. The fertilizer-N and feed-N were calculated as N per ha per year. The total application of pesticides was expressed as kg of active ingredient (a.i.) ha^−1^.

### Farmer survey

To obtain baseline data about RFS and RM farmers and farms, we provided a questionnaire to all farmers involved in the survey described in the previous section. These questionnaires were given to the farmers after harvest (late September). The questionnaire asked farmers about their age, education, farm size, off-farm activities, household assets, use of machines, prices obtained for their products (rice grain and fish), operating costs of rice and fish cultivation, and knowledge of fish and rice cultivation.

Net income was estimated by using data set from farmer survey of RFSs and RMs. The net income was calculated as: total income minus total cost. Total income includes the incomes of rice grain and fish. Total cost includes the costs of fish feed, fish fry, rice seed, chemical fertilizer and pesticides for rice, labor, machine rental and field construction.

### Data analysis

To compare rice yields in RFSs and in their corresponding RMs, we calculated the yield change (%) of rice in RFS vs. RM as: rice yield change (%) = ([yield in RFS-yield in RM]/yield in RM) × 100%. Then, we separated the samples into two groups: samples with positive values of rice yield change (%) and samples with negative values of rice yield change (%). We further compared fish yield and refuge percentage between these two groups.

To estimate the maximum fish yield (referred to as the “threshold yield”) that does not result in a reduction in rice yield, we performed independent polynomial fitting for the relationship between rice yield change (%) and fish yield for each type of intensive RFS (R version 3.2.3)^22^. In this analysis, we fixed the intercept at zero so that the polynomial curves intercepted the axes at coordinates (0, 0), a point at which fish yield is zero in the RFS. We also conducted bootstrapping for 1000 iterations to estimate confidence intervals for the threshold value of fish yield for each RFS. For each bootstrap iteration, we randomly sampled with replacement n times from each RFS data set (n = sample size of the data set) to fit the relationship with a binomial equation and calculated the threshold of fish yield. The lowest and highest 2.5% values were then chosen to represent the lower and higher 95% confidence limits.

With respect to the threshold values for fish yield, simple linear regressions were also used to analyse the relationships between fish yield and refuge percentage. To compare the slopes of regression lines of fish yield and refuge percentage in different RFSs, we conducted an analysis of covariance (ANCOVA) with the types of RFS as categorical predictor variable, refuge percentage as covariate and fish yield as response variable.

We also compared RFSs and RMs for the use of chemical fertilizer-N and pesticides, the prices obtained for the products (rice grain and fish), net income, farm size, and farmer age and education. For each type of RFS, the general linear model (GLM) in R (version 3.2.3)^22^ was also used to perform two-way ANOVAs with location (province) as a random factor and culture type (RM or RFS) as a fixed factor. Before the analysis, data were log-transformed to meet assumptions of normality and homogeneity.

## Additional Information

**How to cite this article**: Hu, L. *et al*. Can the co-cultivation of rice and fish help sustain rice production? *Sci. Rep.* **6**, 28728; doi: 10.1038/srep28728 (2016).

## Supplementary Material

Supplementary Information
