# Supplementary material for: Can the co-cultivation of rice and fish help sustain rice production?
Source: Sci Rep. 2016 Jun 28;6:28728. doi: 10.1038/srep28728 (PMC4923892; doi:10.1038/srep28728)
Supplement: Supplementary Information [file srep28728-s1.pdf]

Can the co-cultivation of rice and fish help sustain rice production?

Liangliang Hu[+,1], Jian Zhang[+,1], Weizheng Ren[+,1], Liang Guo[1], Yongxu Cheng[2], Jiayao Li[2], Kexin Li[3], Zewen Zhu[3], Jiaen Zhang[4], Shiming Luo[4], Lei Cheng[1], Jianjun Tang[1*], and Xin Chen[1*]

[1] College of Life Sciences, Zhejiang University, Hangzhou 310058

[2]College of Aquaculture and Life Sciences, Shanghai Ocean University, Shanghai 201306

[3]National Aquaculture Technical Extension Station, Ministry of Agriculture of People's Republic of China, Beijing 100125

[4]Department of Ecology, South China Agricultural University, Guangzhou 510642, China

* Corresponding author: chen-tang@zju.edu.cnor chandt@zju.edu.cn

+ These authors contributed equally to the work.

Supplementary information

Supplementary Panel 1: the five intensive types of RFSs

The rice-carp system has a long history and is now widely used throughout the rice-growing areas of China[1,2]. In this system, the common carp (*Cyprinus carpio*) has evolved diverse genotypes that are adapted to different rice-field environments (Supplementary Figure 1A). Fish fry are released into the rice field immediately after rice is transplanted and "live" together with rice plants until harvest. Although common carp can grow well by foraging natural food sources in rice fields, fish feed and a refuge (a trench or pit) are required to ensure high fish yields.

The rice-crab system is another common type of RFS (Supplementary Figure 1B)

and is rapidly developing in China[3]. This system uses the Chinese mitten crab, which has evolved two genotypes: one adapted to the climate of northern China and the other adapted to southern China. In the rice-crab system, juvenile crabs are released into the rice field 1 week after rice is transplanted and "live" together with rice plants until harvest. The Chinese mitten crab molts several times during its life cycle and requires a relatively large refuge area (or a low density of crabs) and high quality water within a specific temperature range. In addition, high quality feed is required for high crab yield.

The rice-crayfish system has expanded substantially in southern China over the last 20 years[4]. The crayfish used in this system is *Procambarus clarkii* (Supplementary Figure 1D). Crayfish can live in rice fields throughout the year and can feed on straw, weeds, and macro-algae. Thus, the rice-crayfish system requires only a small quantity of fish feed.

The rice-loach system is a traditional system because loach naturally inhabits rice fields (Supplementary Figure 1E). For high yields of loach, farmers use varieties of the loach *Misgurnus anguillicaudatus*[5]**.** Like carp in the rice-carp system, young loaches are released into the field and remain there until harvest. A refuge and a large quantity of fish feed are required for high yields.

The rice-turtle system uses the Chinese soft-shelled turtle, *Pelodiscus sinensis* (Supplementary Figure C). This new system has developed rapidly over the past 10 years because of the economic value of the turtle, which provides protein and has medicinal effects[4]. The turtles often live in rice fields for 2 years before they are

harvested. From November to May, when there is no rice, the turtles remain in the refuge. High turtle yields require high quality fish feed.

References

1. Li, K.X., Zhu, Z.W. & Qian, Y.L. Trend characteristics and development proposals of a new round rice-fish culture. *Chin. Fisheries Econ.* **29,** 17-21 (2011) (in Chinese).
2. You, X.L. Rice-fish culture: A typical model of sustainable traditional agriculture. *Agr. Archaeol.* **4,** 222–224 (2006) (in Chinese).
3. Xu, M., Ma, X.Z. & Wang, W. Effects of different cultivation patterns on rice yield andcrab in rice-crab culture system. *Agr. Sci. China* **47,** 1828-1835 (2014).
4. Hu, L.L. *et al*. Development of rice-fish system: Today and tomorrow. *Chin. J. Eco-Agric.* **23,** 268-275 (2015) (in Chinese).
5. Lu, W.Q. Economic return from rice-loach system. *Sci. Fish Farm.* **8,** 26-27 (2013) (in Chinese).

Supplementary Figure

Supplementary Figure 1. Diagrams of the five intensive RFSs and the corresponding fish species. A-E indicates the typical field configuration of the five RFSs. The provinces in China where the RFS fields were photographed are indicated in parentheses. **a-e** illustrate the habitat where rice and fish coexist. Detailed information for the five intensive RFSs is provided in WebPanel 1.

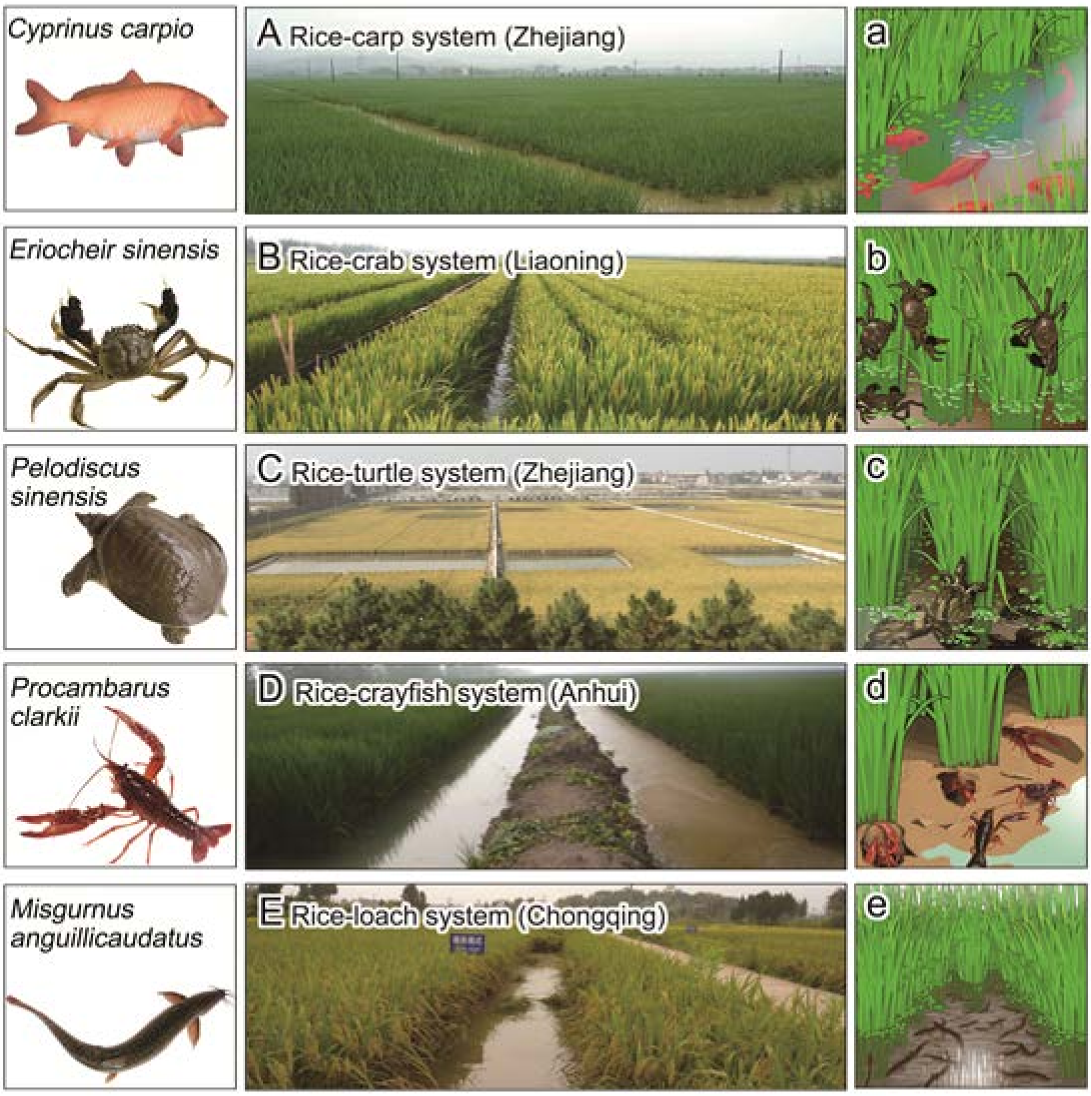

Supplementary Tables

Supplementary Table 1. Equations describing the percentage change in rice yield (relative to the yield in rice monoculture) as a function of fish yield for each type of intensive RFS.

Type of RFS	Equation*	Equation root**	R^2	P
Rice-carp	$y = -5.31x^2 + 11.19x$	0, 2.106	0.6758	0
Rice-crab	$y = -21.78x^2 + 14.36x$	0, 0.657	0.9140	0
Rice-crayfish	$y = -6.24x^2 + 10.4x$	0, 1.681	0.7050	$4.89e^{-8}$
Rice-loach	$y = -5.28x^2 + 8.40x$	0, 1.582	0.6620	$6.28e^{-13}$
Rice-turtle	$y = -1.61x^2 + 5.83x$	0, 3.621	0.7767	$5.59e^{-8}$

*: y is the percentage change in rice yield in the RFS relative to yield in rice monoculture (RM); x is “fish” yield in the RFS.

**: The equation root is that value for fish yield when the percentage change in rice yield is equal to zero. This value for fish yield is the “threshold value”; when the fish yield exceeded this threshold, rice yield was lower in the RFS than in the corresponding RM.

Supplementary Table 2. Equations describing the “fish” yield as a function of refuge percentage for each type of intensive RFS.

Type of RFS	Equation*	R^2	P
Rice-carp	$y = 0.13x + 0.10$	0.56	$9.99e^{-16}$
Rice-crab	$y = 0.03x + 0.23$	0.77	0
Rice-crayfish	$y = 0.05x + 1.04$	0.41	$1.4e^{-4}$
Rice-loach	$y = 0.13x + 0.10$	0.70	$8.44e^{-15}$
Rice-turtle	$y = 0.31x - 0.76$	0.51	$7.83e^{-5}$

*: y is the fish yield; x is refuge percentage in the RFS.

Supplementary Table 3. Results of a statistical analysis comparing rice yield, net income, fertilizer-N, and pesticides in RMs vs. in intensive RFSs in which fish yields did not exceed the threshold. A significant F statistic indicates that rice yield and net income were greater in the RFS than in the RM and that fertilizer-N and pesticide use were lower in the RFS than in the RM.

	Rice-carp	Rice-crab	Rice-crayfish	Rice-loach	Rice-turtle
Rice yield	$F_{1,119}$ = 19.467, P = 0.0001	$F_{1,95}$=4.869, P =0.030	$F_{1,24}$=6.907, P = 0.015	$F_{1,79}$=1.518, P = 0.222	$F_{1,26}$=1.484, P = 0.234
Net income	$F_{1,119}$ = 125.778, P = 0.0001	$F_{1,95}$= 260.384, P = 0.0001	$F_{1,24}$=98.399, P = 0.015	$F_{1,79}$=119.484, P = 0.0000	$F_{1,26}$=106.856, P = 0.0000
Fertilizer-N	$F_{1,119}$ = 51.136, P = 0.000	$F_{1,95}$=13.306, P =0.001	$F_{1,24}$=16.521, P = 0.000	$F_{1,79}$=35.048, P = 0.0.000	$F_{1,26}$=41.206, P = 0.000
Pesticide	$F_{1,119}$ = 179.171, P = 0.0001	$F_{1,95}$=102.123, P =0.001	$F_{1,24}$=232.822, P = 0.000	$F_{1,79}$=114.964, P = 0.0.000	$F_{1,26}$=82.148, P = 0.000

Supplementary Table 4. Examples of identifiable brands of high quality rice or fish products from intensive RFSs.

	Type of RFS	Brand name	Price of products (Chinese Yuan kg^{-1}, mean ± SE)	
**Rice products**			Brand	Local average
	Rice-crab	Crab-rice grain	11±2.0	7±0.8
	Rice-crab	Crab-rice grain	12±1.1	6±1.1
	Rice-turtle	Turtle-rice-grain	30±2.5	5±1.5
	Rice-crayfish	Crayfish rice grain	15±1.8	6±1.0
	Rice-carp	Carp-rice grain	18±2.0	5±1.2
	Rice-carp	Carp-rice grain	12±1.5	5±1.5
**“Fish” products**				
	Rice-carp	Field carp	60±3.5	15±2.0
	Rice-turtle	Black field turtle	84±2.5	70±10.0
	Rice-carp	Red field carp	45±1.8.	20±3.0
	Rice-crab	Field crab	42±1.5	30±2.5
